# First Report of the Emergence of Peste des Petits Ruminants Lineage IV Virus in Senegal

**DOI:** 10.3390/v16020305

**Published:** 2024-02-17

**Authors:** Aminata Ba, Gaye Laye Diop, Mbengué Ndiaye, Michel Dione, Modou Moustapha Lo

**Affiliations:** 1 Institut Sénégalais de Recherches Agricoles, Laboratoire National de l’Elevage et de Recherches Vétérinaires (ISRA-LNERV), Dakar-Hann BP 2057, Senegal; gayelayegld@gmail.com (G.L.D.); mbenguallb@gmail.com (M.N.); moustaphlo@yahoo.fr (M.M.L.); 2 International Livestock Research Institute (ILRI), Dakar 24265, Senegal; m.dione@cgiar.org

**Keywords:** Peste des petits ruminants, lineage IV, molecular characterization, Senegal

## Abstract

Peste des petits ruminants (PPR) is a highly contagious viral disease and one of the deadliest affecting wild goats, sheep, and small ruminants; however, goats are generally more sensitive. The causative agent is the Peste des Petits Ruminants virus (PPRV), which is a single-stranded RNA virus of negative polarity belonging to the Paramyxoviridae family. In February 2020, an active outbreak of PPR was reported in a herd of a transhumant farmer in the village of Gainth Pathé (department of Kounguel, Kaffrine region, Senegal). Of the ten swabs collected from the goats, eight returned a positive result through a quantitative real-time PCR. The sample that yielded the strongest signal from the quantitative real-time PCR was further analyzed with a conventional PCR amplification and direct amplicon sequencing. A phylogenetic analysis showed that the sequence of the PPR virus obtained belonged to lineage IV. These results confirm those found in the countries bordering Senegal and reinforce the hypothesis of the importance of animal mobility between these neighboring countries in the control of PPRV. In perspective, following the discovery of this lineage IV in Senegal, a study on its dispersion is underway throughout the national territory. The results that will emerge from this study, associated with detailed data on animal movements and epidemiological data, will provide appropriate and effective information to improve PPR surveillance and control strategies with a view to its eradication.

## 1. Introduction

In Senegal, the production of small ruminants is an activity of choice for vulnerable populations such as women and young people because of their short reproductive cycle and low investment costs compared to other livestock such as cattle [1], thus offering huge opportunities for reducing poverty. However, the high burden of disease remains a major constraint to small ruminants production. Among these diseases, Peste des Petits Ruminants (PPR) poses the greatest threat to small ruminant livestock. This disease threatens global food security and the rural economy, but its control is complicated, in particular, due to significant animal movements with transhumance and commercial movements in the infected regions [2].

PPR is a highly contagious viral disease and one of the deadliest affecting wild goats, sheep, and small ruminants; however, goats are generally more sensitive. The causative agent is the Peste des Petits Ruminants virus (PPRV), which is a single-stranded RNA virus of negative polarity belonging to the Paramyxoviridae family and the genus of morbilliviruses closely related to the rinderpest virus eradicated successfully worldwide [3]. PPR-related morbidity reaches 100% in extreme situations, with a mortality rate that can reach 90% [4]. Due to the serious impact of the disease on the economy and food security, but also its alarming rate of spread, the World Organization for Animal Health (OMSA) and the United Nations Food and Agriculture (FAO) have chosen PPR as a target for a global eradication program [5]. Based on the molecular characterization of the partial sequence of the N gene, the PPR virus is classified into four genetically different lineages (I, II, III, and IV) [6].

Historically, the geographical distribution of these lineages was distinct from the circulation of lineages I, II, and III in Africa and that of lineage IV in Asian countries and the Middle East [7]. However, several studies have shown the detection of lineage IV in several African countries over the past decades [8,9,10]. In West Africa, lineage I was last officially detected in 1994 in Senegal [11]. However, since that date, no information is available on the molecular characterization of the PPRV. Until 2010, the results of the molecular characterization of circulating strains of the PPR virus obtained with the study by Salami H et al. between 2010 and 2014 showed that only lineage II could be detected in Senegal during this period [12]. The study by Tounkara et al. in 2019 also confirmed that only lineage II was detected in samples taken in 2013 from Dakar markets [13]. Long time well-known in Asia, lineage IV was subsequently discovered in several African countries, particularly countries bordering Senegal, such as Mali [14]. However, until then, lineage IV has never been described in Senegal.

In this study, we reported the detection of the PPRV lineage IV for the first time in Senegal.

## 2. Materials and Methods

### 2.1. Outbreak Investigation and Sample Collection

**Figure 1:**
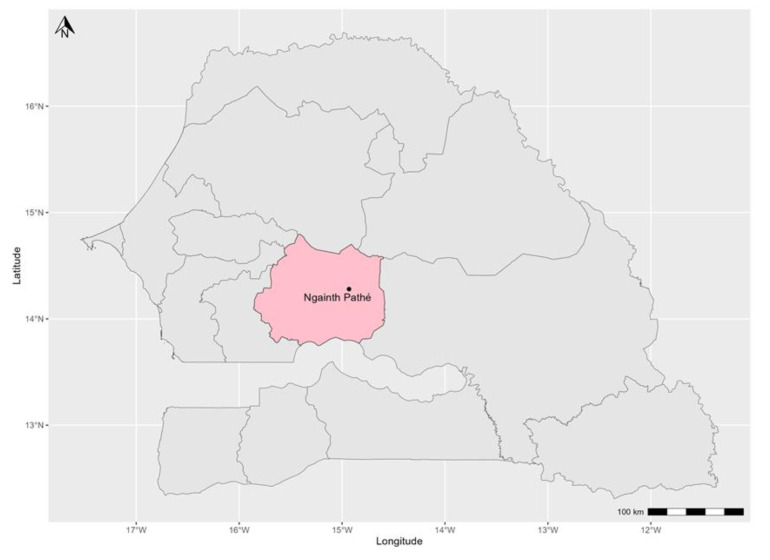
A map of Senegal showing the Kaffrine region (shaded pink) and the locality of Gainth Pathé where the samples analyzed in this study were collected from small ruminants exhibiting signs suggestive of PPRV infection.

In February 2020, an active outbreak of PPR was reported in a mixed herd of 160 sheep and goats of a transhumant farmer in the village of Gainth Pathé (department of Kounguel, Kaffrine region). The transhuman farmer had left the village of Tatki Hillo in the department of Podor (northern Senegal) with his herd at the beginning of November 2020. Along the way, he stayed in the Sylvopastoral zone in Téssékré, department of Linguère, region of Louga before reaching the locality of Gainth Pathé in the central zone in the Kaffrine region (Figure 1), where the samples were collected. Clinical signs observed in infected animals were tearing, coughing, dyspnoea, throwing up, diarrhea, and ulcerations in the gingival mucosa. At the start of the infection, 25 of them presented very intense digestive and respiratory syndromes with high mortality in lambs and kids over 80% of the population. All sick animals were sampled by taking an oculo-nasal for animals with respiratory syndromes or rectal swabs for animals with diarrhea for some types of samples. The swabs were then transported to the laboratory in 2 mL tubes containing 500 µL of viral transport medium and then stored in liquid nitrogen.

### 2.2. RNA Extraction and PCR

Of the ten (10) swabs collected from goats, the viral RNA was extracted using the QIAGEN RNeasy mini kit according to the supplier’s instructions. The extracted RNAs were then analyzed with reverse transcriptase quantitative real-time (RT-qPCR) using the qScritpt XLT One step RT-qPCR Tough mix amplification kit from Quantabio with the sets of primers and probes developed by Batten et al. [15] in order to estimate the viral load of the samples. All samples yielding cycle threshold (CT) values less than ≤30 were subjected to further analysis with conventional PCR using forward primer NP3 (5′-GTCTCGGAAATCGCCTCACAGACT-3′) and reverse primer NP4 (3′-CCTCCTCCTGGTCCTCCAGAATCT-5′) [16] located at the 3′ end of the N gene using the One Step RT-PCR mix kit from Qiagen, making it possible to amplify a segment of 351 base pairs (bp) of the PPRV gene. The PCR was carried out according to the following program: 1 cycle RT (Retro transcription): 30 min at 50 °C, 1 cycle of primer hybridization: 15 min at 95 °C and 40 cycles of elongation (15 s 95 °C 45 s at 60 °C; 45 s at 72 °C). The size of the amplified fragments was visualized after electrophoretic migration of the PCR products in a 1.5% agarose gel with 1X TAE buffer.

### 2.3. Sequencing and Editing

The amplification products were then purified using the PROMEGA SV Gel and PCR clean-up system-50rxn wizard kit (Madison, WI, USA). Amplicons thus purified were sent to LGC genomics (Berlin, Germany) for sequencing using the Sanger method. The analysis of the generated sequences was carried out with the Molecular Evolutionary Genetics Analysis 7 (MEGA 7) software version 7.0.26 [17]. The Bioedit software version 7.0.5.3 [18] made it possible to clean the sequences and carry out the multiple alignments of the N gene of the sample with other PPRV sequences representative of the four genetic lines obtained in Genbank. The two-parameter Kimura model proposed by the MEGA 7 program was used. From this model, phylogenetic trees were constructed in MEGA 7 using the likelihood method (ML) with a repetition of 1000 (bootstraps).

### 2.4. Nucleotide Sequence Accession Number

The genome sequence of the GTP_SEN-KAFF2021 PPRV strain is available at Genbank under the accession number OR819867.

## 3. Results

Almost all affected animals expressed very overt digestive and respiratory syndromes associated with fetid diarrhea, mucopurulent discharge, and intense lacrimation. Then, in the final phase, a high lethality of up to 80% of the affected herd was observed. Goat kids had a mortality rate of up to 100%, while morbidity also approached 100% for adults who survived.

**Figure 2:**
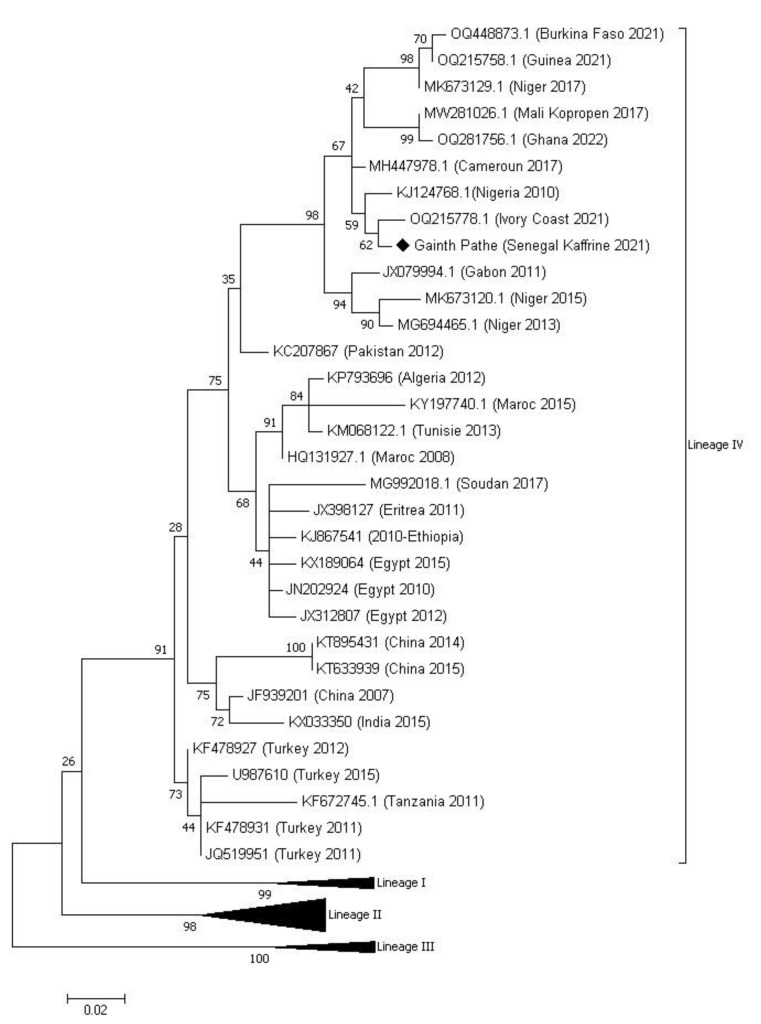
Phylogenetic tree from the partial sequences of the N gene of the PPRv constructed using the likelihood method (ML) following the two-parameter Kimura model. The sample isolated in this study is indicated on the tree with a black diamond with the name of the sampling location (Gainth Pathé), the country (Senegal), and the year of sampling (2021). Numbers at nodes are Bootstrap values obtained from 1000 repetitions. The sequences of lineages I, II and III have been collapsed in this figure for greater clarity.

Ten swabs (10) taken were analyzed, and eight (8) came back positive through real-time PCR (Table S1). The sample with the lowest CT value, 18.38, was further analyzed using RT-PCR amplification with the NP3/NP4 primer pair, and then the purified amplicon was sent for sequencing. Phylogenetic analysis showed that the sequence of the PPR virus obtained belonged to lineage IV (Figure 2).

## 4. Discussion

This is the first time that this lineage IV has been reported in Senegal. This new epidemiological situation of PPR in Senegal was predictable given the epidemiological context of PPR prevailing in neighboring countries and animal mobility. The phylogenetic tree shows the identification of several distinct genetic subclades within lineage IV, which can be considered as part of different transmission networks evolving in parallel in West Africa. The Kaffrine Gainth Pathe sequence from this study fits into one of these subclades in a cluster with 98% lymph node support bringing together PPR lineage IV virus sequences identified in 2021 in Ivory Coast, Burkina Faso, and Guinea [19], 2010 in Nigeria [10], 2017 in Mali, Cameroon and Niger [14,20] and 2022 in Ghana [19]. These results confirm the conclusions drawn from the study by Tounkara et al. in 2021 on the risk of introducing lineage IV into Senegal due to animal mobility between Senegal and Mali [14]. Indeed, Mali is the largest exporter of animals during Muslim holidays to cross-border countries. In the same dynamic, the similarity of the sequences from Nigeria and Niger had already been described by Souley et al. and Mantip et al. [20,21], suggesting a cross-border movement of PPR between these two neighboring countries and beyond towards Cameroon. This explains this grouping of these sequences belonging to these countries in the same cluster. Furthermore, the phylogenetic tree also shows within lineage IV geographical differences between the viruses identified in West Africa, including the sequence of our study (Gainth pathe Kaffrine 2021), the viruses identified in North Africa (Morocco 2015, Tunisia 2013, Egypt 2010, etc.) and the viruses identified in Asia (China 2015, India 2015, etc.). These geographical differences had already been described previously by other past studies [9,21] but also more recently with the results of the study by Couacy–Hymann et al. [19], which showed the first description of lineage IV of the PPR virus in four West African countries (Ivory Coast, Guinea, Burkina Faso and Ghana). These results highlight the importance of animal mobility in the transmission of PPR in the region. With these results, it would be ideal to combine genetic data with mobility networks to identify key sites of entry and spread of the virus in specific areas. Such information could strengthen our ability to develop control and surveillance strategies adapted to local conditions, using, among other risk factors, information on animal mobility. Our study once again reinforces the hypothesis that lineage IV became the predominant lineage in West Africa, as previously demonstrated in other studies [9,13,19].

Although phylogenetic analyses based on partial sequences of the N gene have provided very useful information on the genetic characterization of the Peste des petits ruminants virus, it would, however, be important to sequence the complete genome of this lineage in order to achieve more informative and in-depth analyses. Indeed, analyses based on the complete genome with a larger number of samples covering the country, associated with information on animal movements and epidemiological data, would make it possible to understand the phylogenetic relationships within PPRV lineage IV and the dynamics of its transmission in Senegal and beyond even in West Africa.

## 5. Conclusions

We report the first detection of lineage IV of the PPRV in an outbreak in Gainth Pathé village (Kaffrine region) in Senegal in February 2021. Phylogenetic analysis showed that the sequence found during this outbreak is genetically linked with sequences found in Ivory Coast, Mali, Niger, Nigeria, Guinea, Burkina Faso, Ghana and Cameroon. These results confirm those found in the latter countries and reinforce the hypothesis of the importance of animal mobility between these neighboring countries.

In perspective, following the discovery of this lineage IV in Senegal, a study on its dispersion is underway throughout the national territory. The results that will emerge from this study, associated with detailed data on animal movements and epidemiological data, will provide appropriate and effective information to improve PPR surveillance and control strategies with a view to its eradication.

## Data Availability

Data are contained within the article and the supplementary material.

## Supplementary Materials

The following supporting information can be downloaded at: https://www.mdpi.com/article/10.3390/v16020305/s1, Table S1: RT-qPCR results for the samples analysed.

## References

[1] (2004). Bureau d’Analyse Macro-Economique (BAME), ISRA: Nouvelle Initiative Sectorielle pour le Développement de l’Elevage, Situation et Perspective du Sous-Secteur de L’élevage. https://www.bameinfopol.info/IMG/pdf/NISDEL.pdf.

[2] Fakri F., Embarki T., Parida S., Bamouh Z., Jazouli M., Mahapatra M., Tadlaoui K., Fassi-Fihri O., Richardson C.D., Elharrak M. (2016). Re-emergence of Peste des Petits Ruminants virus in 2015 in Morocco: Molecular characterization and experimental infection in Alpine goats. Vet. Microbiol..

[3] Pastoret P.P., Yamanouchi K., Mueller-Doblies U., Rweyemamu M.M., Horzinek M., Barrett T. (2006). Rinderpest—An old and worldwide story: History to c. 1902. Rinderpest and Peste des Petits Ruminants.

[4] Kumar N., Maherchandani S., Kashyap S.K., Singh S.V., Sharma S., Chaubey K.K., Ly H. (2014). Peste des Petits Ruminants Virus Infection of Small Ruminants: A Comprehensive Review. Viruses.

[5] OIE, FAO Global Control and Eradication of PPR. http://www.oie.int/eng/PPR2015/doc/PPR-Advocacy-EN.pdf.

[6] Kwiatek O., Minet C., Grillet C., Hurard C., Carlson E., Karimov B., Diallo A. (2007). Peste des Petits Ruminants (PPR) outbreak in Tajikistan. J. Comp. Pathol..

[7] Banyard A.C., Parida S., Batten C., Oura C., Kwiatek O., Libeau G. (2010). Global distribution of peste des petits ruminants virus and prospects for improved diagnosis and control. J. Gen. Virol..

[8] Kwiatek O., Ali Y.H., Saeed I.K., Khalafalla A.I., Mohamed O.I., Obeida A.A., Libeau G. (2011). Asian lineage of peste des petits ruminants virus, Africa. Emerg. Infect. Dis..

[9] Dundon W., Diallo A., Cattoli G. (2020). Peste des petits ruminants in Africa: A review of currently available molecular epidemiological data, 2020. Arch. Virol..

[10] Woma T.Y., Adombi C.M., Yu D., Qasim A.M., Sabi A.A., Maurice N.A., Olaiya O.D., Loitsch A., Bailey D., Shamaki D. (2016). Co-circulation of Peste des Petits Ruminants Virus Asian lineage IV with Lineage II in Nigeria. Transbound. Emerg. Dis..

[11] Tounkara K., Bataille A., Adombi C.M., Maikano I., Djibo G., Settypalli T.B.K., Loitsch A., Diallo A., Libeau G. (2018). First genetic characterization of Peste des Petits Ruminants (PPR) from Niger: On the advancing front of the Asian virus lineage. Transbound. Emerg. Dis..

[12] Salami H., Croville G., Kwiatek O., Mariette J., Klopp C., Valiere S., Libeau G. (2014). Complete genome sequence of a field strain of peste des petits ruminants virus isolated during 2010–2014 epidemics in Senegal. Genome Announc..

[13] Tounkara K., Kwiatek O., Niang M., Abou Kounta Sidibe C., Sery A., Dakouo M., Bataille A. (2019). Corrigendum: Genetic Evidence for Transboundary Circulation of Peste des Petits Ruminants across West Africa. Front. Vet. Sci..

[14] Tounkara K., Kwiatek O., Sidibe C.A.K., Sery A., Dakouo M., Salami H., Lo M.M., Ba A., Diop M., Niang M. (2021). Persistence of the historical lineage I of West Africa against the ongoing spread of the Asian lineage of peste des petits ruminants virus. Transbound. Emerg. Dis..

[15] Batten C.A., Banyard A.C., King D.P., Henstock M.R., Edwards L., Sanders A., Buczkowski H., Oura C.C., Barrett T. (2011). A real time RT-PCR assay for the specific detection of Peste des petits ruminants virus. J. Virol. Methods.

[16] Couacy-Hymann E., Roger F., Hurard C., Guillou J., Libeau G., Diallo A. (2002). Rapid and sensitive detection of peste des petits ruminants virus by a polymerase chain reaction assay. J. Virol. Methods.

[17] Tamura K., Stecher G., Peterson D., Filipski A., Kumar S. (2023). MEGA7: Molecular Evolutionary Genetics Analysis Version 7.0.26. Mol. Biol. Evol..

[18] Hall T.A. (1999). BioEdit: A user-friendly biological sequence alignment editor and analysis program for Windows 95/98/NT. Nucleic Acids Symp. Ser..

[19] Couacy-Hymann E., Berete K., Odoom T., Zerbo L.H., Mathurin K.Y., Kouakou V.K., Doumbouya M.I., Balde A., Ababio P.T., Ouoba L.B. (2023). The Spread of Peste Des Petits Ruminants Virus Lineage IV in West Africa. Animals.

[20] Souley M.M., Ibrahim A.S., Sidikou D., Dundon W.D., Cattoli G., Abdou A., Soumana F., Yaou B. (2019). Molecular epidemiology of peste des petits ruminants in Niger: An update. Transbound. Emerg. Dis..

[21] Mantip S., Sigismeau A., Shamaki D., Woma T.Y., Kwiatek O., Libeau G., Farougou S., Bataille A. (2022). Molecular epidemiology of peste des petits ruminants virus in Nigeria: An update. Transbound. Emerg. Dis..

