# Supplementary material for: First Report of the Emergence of Peste des Petits Ruminants Lineage IV Virus in Senegal
Source: Viruses. 2024 Feb 17;16(2):305. doi: 10.3390/v16020305 (PMC10892897; doi:10.3390/v16020305)
Supplement: Supplementary file 1 [file viruses-16-00305-s001.zip › viruses-2761316-supplementary.pdf]

Table S1: RT-qPCR results for the samples analysed

Number of order	Nature of sample	Cq values	Sample status
1	Oral and nasal Swab	NA	Negative
2	Oral and nasal Swab	23.99	Positive
3	Oral and nasal Swab	25.50	Positive
4	Oral and nasal Swab	28.70	Positive
**5**	**Oral and nasal Swab**	**18.38**	**Positive**
6	Oral and nasal Swab	28.81	Positive
7	Oral and nasal Swab	22.63	Positive
8	Anal Swab	NA	Negative
9	Anal Swab	25.20	Positive
10	Anal Swab	19.34	Positif
